# A case of chimerism-induced paternity confusion: what ART practitioners can do to prevent future calamity for families

**DOI:** 10.1007/s10815-017-1064-6

**Published:** 2017-10-23

**Authors:** Kayla M. Sheets, Michael L. Baird, Julie Heinig, Debra Davis, Mary Sabatini, D. Barry Starr

**Affiliations:** 1 Vibrant Gene Consulting, LLC, PO BOX 390752, Cambridge, MA 02139 USA; 2 DNA Diagnostics Center, Inc., One DDC Way, Fairfield, OH 45014 USA; 3 Reproductive Endocrinology and Fertility, Massachusetts General Hospital, 55 Fruit St, Boston, MA 02114 USA; 4 Department of Genetics, Stanford University School of Medicine, Stanford, CA 94305-5120 USA

**Keywords:** Congenital chimera, Tetragametic chimerism, Semen sample mix-up, Gamete mix-up, Assisted reproductive technology, False negative DNA paternity testing

## Abstract

In the fertility clinic setting, a negative DNA paternity test result usually suggests a sample mix-up likely occurred at the testing company or in the clinic. However, we report a case where, despite repeat negative paternity test results, the alleged father (referred to as “the proband”) was confirmed to be the baby’s father. The proband, a 34 year-old male, contacted our research group when routine blood testing revealed discrepant blood types between the parents and the baby, repeat paternity tests were negative (excluding the proband as the baby’s father), and the fertility clinic found no evidence of any wrongdoing. Microarray technology was utilized to confirm biological relatedness, which revealed an avuncular (uncle/nephew) relationship. Additional tissue samples were analyzed and family studies were conducted at paternity and forensic laboratories using STR-based DNA tests to elucidate the proband’s condition of *congenital tetragametic chimerism*. His paternity was subsequently affirmed and the fertility clinic exonerated of claims of a semen sample mix-up. This case underscores the possibility that some allegations of fertility clinic missteps may be explained by undiagnosed *chimerism,* a condition where an individual harbors two distinct genomes. We offer specific suggestions for improving laboratory reporting and creating clinical guidelines to aid in identifying and rectifying future cases of false exclusions of paternity due to chimerism.

**Electronic supplementary material:**

The online version of this article (10.1007/s10815-017-1064-6) contains supplementary material, which is available to authorized users.

## Introduction

Parental (paternity/maternity) DNA testing is indicated where a parent-child relationship requires confirmation. Common reasons for testing are child custody, child support, and immigration cases. In the USA, well over 382,000 legal paternity tests are ordered annually. (With less than 60% of all surveyed paternity testing laboratories reporting data to the American Association of Blood Banks [AABB] in 2010, this is a considerable underestimate.) Additionally, an unknown number are self-administered at home.

Currently, the gold standard technology used for these tests is a PCR-based assay that amplifies short tandem repeat (STR) regions within the tested person’s DNA [1]. This technology was developed in the 1980s and has known limitations and failures [2]. Understanding these is of critical importance. A negative result, where the proband is *excluded* as the father of the child, has serious consequences to families ranging from broken trust (fidelity concerns), divorce, lost child support, or denied immigration. In cases where a baby was conceived in a fertility clinic, false negative tests can have ramifications for the clinic and staff.

In this paper, we present a case where the gold standard STR-based DNA paternity test repeatedly excluded the proband from being the biological father, despite the fact that the child was conceived by a semen sample provided by the proband. Unbeknownst to the family and to the fertility clinic, the reason for the test’s false negative result was that the father harbored more than one genome, a condition called *chimerism*.

The specific form of congenital chimerism in our case study was determined to be *tetragametic chimerism* (tetra = four gametes). Such chimerism can occur when cells from dizygotic twin embryos fuse early in development leading to the birth of a singleton with two cell lines containing two different genomes [3]. If a male with tetragametic chimerism conceives a child with a sperm cell that contains a genome distinct from that of the tested paternal tissue (usually buccal or peripheral blood), standard paternity DNA tests will report non-paternity. Through follow-up DNA testing, the genetic relationship between the proband and the child can be more accurately depicted as *avuncular*. After all, the DNA of the proband’s tested tissue is more similar to the child’s *uncle*, not father.

The *Guidance for Standards for Relationship Testing Laboratories* (published by the Relationship Testing Program Unit of the American Association of Blood Banks) currently does not have provisions that look for avuncular relationships in standard PCR-based paternity tests. Therefore, current guidance does not safeguard against false negative results for chimeric parents. Moreover, since chimerism poses little or no threat to the individual’s health, and since most chimeras do not display associated traits, the majority of existing chimeras are believed to be undiagnosed [3, 4]. As a result, an unknown number of chimeras are at risk of being *falsely excluded* from being the biological parents of their children by the current gold standard PCR-based paternity tests.

Below, we present the details of the studied case. To the best of our knowledge, this is the first confirmed case of a father with tetragametic chimerism having a child with the genome originating from his unborn twin. In other words, this is the first confirmed case of a man fathering his unborn brother’s child and so, in essence, fathering his nephew.

We suggest improvements to current laboratory standards that take into account chimerism and reduce the risk of false paternity exclusions for chimeric individuals. We provide clinical suggestions for assisted reproductive technology (ART) practitioners, in the event of accusations of mistakes and wrongdoing. We provide clinical guidelines for when to suspect undiagnosed chimerism as a cause of purported false relationship DNA test results, and when to consider contacting a genetic expert.

### About chimerism

Chimerism is a condition where an individual carries more than one complete genome. Chimerism can be acquired or congenital. Acquired chimerism can arise in multiple ways.

Low levels of chimerism can be acquired via blood transfusions, or bone marrow or organ transplants, when cells from the donor become incorporated in the recipient’s body. In women, acquired chimerism has been detected after pregnancy, when embryonic cells from the fetus circulate and colonize tissues of the mother’s body. Maternal-fetal microchimerism may in fact be the most common form of chimerism found in humans [5, 6]. Such chimerism could occur even if the pregnancy did not survive to recognition [3].

Congenital chimerism can arise through various mechanisms. Blood-exclusive chimeras can result from blood vessel anastomoses between dichorionic placentas. Extensive testing of additional tissues is required to diagnose patients with chimerism exclusive to blood. This testing is often not feasible, leading to speculation that these individuals may have additional tissues involved. [7] Fertilization of an egg and associated polar body by two sperm, followed by their subsequent fusion can lead to chimerism. Tetragametic chimerism occurs when cells from dizygotic twin embryos fuse early in development leading to the birth of a singleton with two cell lines containing two different genomes.

This case study involves diagnosing an individual with tetragametic chimerism, who unknowingly had what we refer to as a major and minor genome. The *major genome* is defined as the chimera’s predominant genome, while the *minor genome* is defined as the less predominant genome which presumably originated from the DZ twin.

### Prevalence of tetragametic chimerism in the era of fertility treatments

The prevalence of tetragametic chimerism remains largely unknown. Most tetragametic chimeras do not display any associated traits. Moreover, no public-wide screening program exists to diagnose chimerism since it poses little risk to the individual’s health. As a result, the majority of existing tetragametic chimeras remain undiagnosed throughout their lifetime, which makes the prevalence of their condition unknown.

Well-established data indicates that one in eight singleton pregnancies originated as a dizygotic twin pregnancy with no evidence of a twin history [8]. It is possible this figure is an underestimate due to the increased use of assisted reproductive technologies. Such pregnancies have the chance of resulting in tetragametic chimeras, but no data exists how frequently this occurs.

Various methods employed by ART are known to increase the rate of multiple pregnancies (e.g., mono and dizygotic twins, triplets). Dizygotic multiple pregnancies are believed to be the leading cause of tetragametic chimerism. ART can also increase the likelihood of monochorionic dizygotic twinning (MC DZT) [9], with five such cases reported in Japan in 2004 alone [10]. Notably, this finding was reported to be an underestimate of the prevalence of MC DZT cases caused by ART. These cases were detected because of sex chromosome discordance discovered in blood samples (thought to be confined blood chimerism). Thus, the cases involving concordant sex chromosomes were missed.

## Results

### Methods summary

#### Ancestry DNA testing

Kits were ordered for the presumed genetic father (proband) and child from 23andMe which utilizes the Illumina HumanOmniExpress-24 format chip. The biological relationship was assessed using the relationship finder application.

#### Confirmatory paternity and forensic DNA testing

Buccal samples were obtained from the mother, child, and proband as well as from the parents of the proband. At DNA diagnostic center (DDC), DNA was extracted from the buccal swabs using organic extraction. Additionally, a semen sample, a blood sample, and skin swabs were collected from the alleged father and DNA was isolated using a differential extraction method. DNA analysis was performed using an Applied Biosystems AmpFlSTR Identifiler® PCR Amplification Kit, a 310 genetic analyzer and Gene Mapper ID v 3.2. The forensic lab was asked to analyze blood and semen samples as they use the more sensitive genetic analyzer, an ABI 310, as opposed to the ABI 3730 used by the paternity testing lab. All other samples were analyzed in DDC’s paternity testing lab.

Our group received the proband’s formal consent to publish his case.

### Identifying the avuncular relationship

The proband, a 34 year-old Caucasian male, presented to our group and later revealed he had patchy bi-toned skin. He and his wife were diagnosed with idiopathic infertility and reported no clinical history of therapeutic transplantation or transfusion. The couple conceived their second son via intrauterine insemination (IUI). At birth, the baby’s blood type (AB+) was found to be inconsistent with the blood types of the proband and his wife (A+ and A−, respectively). The couple contacted the fertility clinic with concerns of a possible mistake. The clinic launched an internal investigation and concluded the proband was the sole Caucasian semen donor that day.

Seeking answers, the couple pursued at-home DNA paternity testing. This testing utilized PCR amplification of the standard 15 STR markers from buccal-derived DNA samples. The test determined that 12 of 15 markers matched, but yielded a probability of paternity of 0%, *excluding* the proband from being the baby’s father.

Because of concerns that the lab or clinic may have made an error, the couple contacted an attorney, who facilitated the procurement of a legal paternity test. The second test, conducted at a different lab, concluded that 11 of 15 alleles matched, but again *excluded* the proband from being the biological father of the child. Both labs queried the same STR markers and differed in the number that matched, and neither paternity test gave any indication of the possibility of a second degree or more distant biological relationship between the proband and the child.

**Fig. 1:**
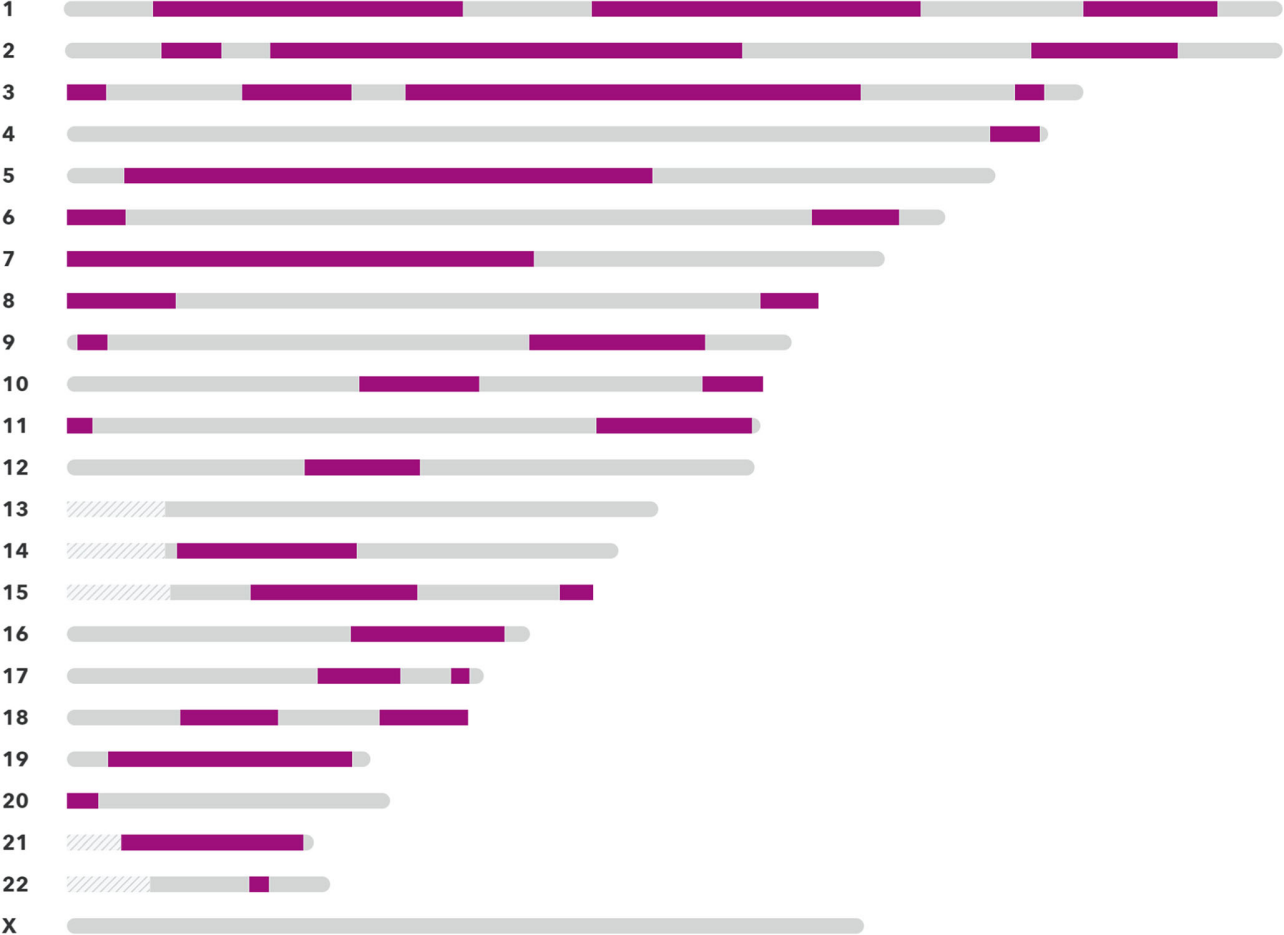
At-home DNA relationship testing result. Ancestry test result depicts chromosomes, shown by gray bars, and ordered according to autosomal number. The green regions represent segments of contiguous SNPs that are shared between the proband and child. In a typical parent/child relationship, the green regions would have longer contiguous green lines across the length of chromosomal segments, rather than short green lines that represent the ~ 25% of shared DNA, as is seen in this uncle/nephew biological relationship

The senior author (Starr) was contacted, and at his recommendation, the proband and child pursued relationship DNA testing that utilized single nucleotide polymorphism (SNP) microarray technology to determine if there was a biological relationship that had been overlooked. Testing was performed by 23andMe, using the Illumina HumanOmniExpress-24 chip, querying over 700,000 markers. This testing and subsequent use of the relative finder feature identified an *avuncular* (uncle/nephew) relationship, demonstrating ~ 25% shared DNA homology (Fig. 1) between the proband and child. This led Starr to suspect that the proband could be a chimera.

The primary author (Sheets) recommended confirmatory testing for the proband and facilitated testing at DNA Diagnostics Center (DDC). The proband provided several different tissue samples and proceeded with testing buccal samples from his wife, his parents, and his two sons (Table 1).

**Table 1:** Proband’s familial studies. The proband’s immediate family members provided buccal samples for STR-based DNA testing at DDC’s relationship testing laboratory. The *major genome* is defined as the chimera’s predominant genome, while the *minor genome* is defined as the less predominant genome, which presumably originated from the DZ twin. The first son’s DNA testing revealed the presence of the proband’s major genome. Meanwhile proband’s second son’s DNA showed the presence of the minor genome and correlated to the proband’s semen-derived DNA sample

Relatives	Tissue type	DNA test method	Result
Wife	Buccal	15 STR	All alleles accounted for in sons
1st son	Buccal	15 STR	Major genome
2nd son	Buccal	15 STR	Minor genome
Proband’s father	Buccal	15 STR	All alleles accounted for in major and minor genome of proband
Proband’s mother	Buccal	15 STR	All alleles accounted for in major and minor genome of proband

Elucidating the cause of the proband’s false negative standard DNA paternity tests and avuncular relationship to his son required confirmatory testing and custom laboratory interpretations. First, the proband’s additional tissue samples were tested to confirm the suspected diagnosis of chimerism. Next, confirmatory testing was required to identify the origin of the proband’s additional alleles. Last, the laboratory devised an algorithm to calculate the probability of paternity using the proband’s minor genome.

### Confirmatory testing: diagnosing the proband

DDC extracted DNA from each of the proband’s tissue samples (blood, semen, hair, skin, and buccal). Two specialized laboratories at DDC were used to analyze the samples via STR-based assays, a paternity lab and a forensic lab. Paternity testing was conducted on all samples. Additional forensic testing was performed where the paternity testing was inconclusive.

In most samples, the paternity lab detected the presence of one of the following: a major genome, a minor genome, or two genomes (Table S1). Again, we define the *major genome* as the chimera’s predominant genome and the *minor genome* as the less predominant genome (which presumably originated from the DZ twin.) The blood and semen samples required testing in the forensic lab as the standard paternity testing assays failed due to the limited amount of DNA present.

This testing successfully identified two distinct cell lines in the proband’s semen. DDC estimated that the minor genome, the source of the DNA for the proband’s second child, comprised about 10% of the semen sample. Forensic testing was able to detect only the major genome in the proband’s blood, though evidence suggested that a second genome may be present. A recent blood typing analysis revealed the proband has predominantly A+ but also detected AB blood, indicating it is likely both genomes are present in blood. Presumably, this recent serum test was a more sensitive analysis than what had been used in prior years, implementing forward and back typing.

The proband’s buccal sample was evaluated in several commercial laboratories, using different DNA testing methodologies (Table 2). Standard STR testing at two paternity laboratories (Genex Diagnostics, DDC) did not detect the proband’s minor genome. STR testing in a forensic laboratory (DDC) also only detected the major genome. Testing at an ancestry laboratory (23andMe) did not detect the minor genome in the proband but elucidated a second-degree relationship with the proband’s second child.

**Table 2:** Comparing methodologies: testing outcomes of Proband’s Buccal DNA. The proband’s buccal-derived DNA sample was analyzed with three different molecular genetic testing platforms. Each methodology yielded varying conclusions. Standard STR-based analysis in paternity laboratories excluded the proband from being the child’s father, providing no indication of a more distant blood relationship. The forensic laboratory utilized a more sensitive protocol, and only detected the proband’s major genome. The SNP-based array used by the ancestry testing laboratory identified a second-degree (avuncular) relationship between the proband and child

Paternity testing (15 STRs)	Forensic testing (15 STRs)	Ancestry testing (SNPs)
Exclusion	Major genome detected	Second-degree relationship

Overall, the confirmatory testing at DDC ascertained that the proband was a chimera, whose sperm, among other tissues, contained two distinct genomes.

### Confirmatory testing: family members

Further testing was conducted to better understand the origin of the proband’s alleles and how they could be used to investigate his paternity.

DNA from the proband’s wife was used to identify maternally inherited alleles in his second child. Through the process of elimination, this allowed the identification of alleles that this son likely inherited from the proband’s minor genome. This data helped enhance the accuracy of the subsequent paternity test probability estimation and interpretation.

DNA samples from the proband’s parents were used as controls. Each allele in the proband needed to be accounted for in his mother and father (Figure S1 and Table S2). This study confirmed that no external DNA had contaminated the proband’s tissue samples, further strengthening the conclusion that the proband contained two closely related genomes.

### Confirmatory testing: resolving the proband’s paternity

DDC confirmed paternity of the proband for both of his sons using paternity and forensic DNA tests. Paternity testing of buccal-derived DNA from the proband and his first son *did not exclude* the proband as the father (data not provided). Forensic testing of the proband’s semen and his second son’s buccal sample *did not exclude* the proband as the father (Table 3). This was consistent with the hypothesis that the proband had tetragametic chimerism and had fathered each son with a different genome.

**Table 3:** Forensic DNA testing of proband’s semen confirms paternity of the second son DDC DNA test report. The proband’s semen contains two cell lines with multiple alleles present at each locus. The combined paternity index (CPI) of 134,323 was calculated using minor alleles

		Mother (buccal)		Child (buccal)		Alleged father (semen)			
Locus	PI	Allele sizes		Allele sizes		Allele sizes			
D3S1358	1.88	15	16	15			15	18	
vWA	1.21	18	19	17	19	16	17	19	
D16S539	1.41	12	13	12	13	11	12	13	
CSF1PO	1.10	11	12	11		10	11	12	
TPOX	0.62	8	9	8		8	9	11	
D8S1179	3.36	10	14	10	15	11	13	15	
D21S11	10.79	29	33.2	27	33.2	27	29	33.2	
D18S51	5.88	15	17	15	19	15	16	19	22
D19S433	1.70	13	14	13	14		13	14	
TH01	1.64	6	8	6	9.3		6	9.3	
FGA	2.36	20	24	20	24	20	23	24	
D5S818	1.37	11	12	11	12		11	12	
D13S317	4.47	8	12	8	13		12	13	
D7S820	2.41	9	10	9	11		9	11	
D2S1338	2.98	19		19		17	19	22	
Amelogenin		X		X	Y		X	Y	
Interpretation:									
Combined paternity index:	134,323			Probability of paternity: 99.9993%					
The alleged father is not excluded as the biological father of the tested child. Based on testing results obtained from analyses of the DNA loci listed, the probability of paternity is 99.9993%. This probability of paternity is calculated by comparing an untested, unrelated, random individual of the Caucasian population (assumes prior probability equals 0.50).									

### Families with chimerism present complex relationships

**Fig. 2:**
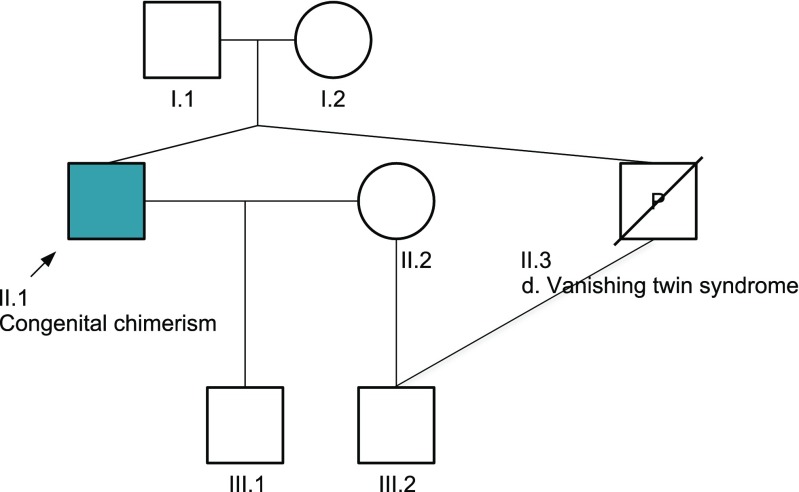
Proband’s pedigree. Tetragametic chimerism gives rise to complex family pedigrees. The proband (II.1) was predicted to have originated as a dizygotic twin pregnancy. Cells from his unborn twin (II.3) were incorporated into his gametes, giving rise to sperm of two distinct cell lines. As a result, the proband’s second son (III.2) was conceived via sperm that contained the minor genome, originally from the vanished twin, II.3

This case illustrates the unusual relationships that arise within families where one parent has tetragametic chimerism (Fig. 2). The proband’s second child was conceived via sperm that contained the proband’s minor genome, which originated from the proband’s unborn twin (II.3). As a result, the proband (based on his major genome) is a *second* degree relative to this son (III.2), thus they are more similarly related as an uncle/nephew. However, based on his minor genome, the proband is also the father. The two children (III.1 and III.2) are neither full nor half-siblings, but are related to some degree in between, sharing an estimated 37.5% of their genes.

## Discussion

This confirmed case of tetragametic chimerism raises many interesting points to consider while interpreting a purportedly negative DNA paternity test.

One of the most impactful consequences of this case study is to point out that some traditional paternity tests which have resulted in negative outcomes (the tested parent was *excluded* as the biological parent) may have been wrong, because the alleged parent may have undiagnosed chimerism. It also points to the importance of follow-up testing in such cases. Relatively inexpensive tests are readily available to easily and reliably distinguish between a parent/child and an avuncular relationship.

Next, we provide recommendations for clinicians and relationship testing labs to prevent future failed parental DNA tests.

### Clinical guidance for assisted reproductive technology practitioners

This unique case presents several guidance points for ART practitioners in terms of evaluating physical findings, interpreting negative parental DNA tests, and facilitating further DNA screening for chimeric individuals.

Most individuals with tetragametic chimerism *do not* display physical traits related to their condition. However, previously published cases were characterized with one or more of the following manifestations: discordant blood types, patches of different colored hair, different colored eyes, or bicolored skin pigmentation which may display lines of Blaschko (Table 4). Disorders of sexual development (DSDs) can be signs of XX/XY chimerism, though most individuals with DSDs will not present with chimerism. It remains unknown as to which traits are most and least prevalent in chimeric individuals.

**Table 4:** Physical traits associated with chimerism. Most individuals with chimerism do not manifest physical traits indicative of their condition, and as a result, are believed to remain undiagnosed. Previous cases have described one or more physical traits listed above, which are associated with the phenotype of blood-exclusive or tetragametic chimerism

Physical trait	Type of chimerism
Discordant blood types	Blood-exclusive or tetragametic
Patchy, different colored hair	Tetragametic
Different colored eyes	Tetragametic
Bicolored skin pigmentation (depicting lines of Blaschko)	Tetragametic
Disorders of sexual development	Tetragametic

DNA paternity tests that rely on STR methodologies are likely to be insufficient for undiagnosed chimeric individuals, as they will not necessarily identify avuncular relationships. Moreover these tests may yield different outcomes, depending on the origin of the DNA samples and on the technology utilized. Had the proband only pursued standard DNA paternity testing, he would have had to concede to a false negative result, with tremendous ramifications for him and for his family.

In the event of a purported failed paternity test, it may be in everyone’s interests for the fertility clinic to determine whether the father is an uncle or unrelated to the child using a microarray-based DNA test as was used in this case. Refer to Fig. 1 as an example of SNP-based microarray results. These tests can simply and reliably identify the avuncular relationships which can result from a parent with tetragametic chimerism.

If the man and the child appear unrelated via the microarray-based test, then paternity issues and/or fertility clinic mistakes should be followed up on. If, on the other hand, the test shows an avuncular relationship, then the parents and/or clinic may want to consider additional testing to determine if the father is indeed a chimera. Given the complexity of the situation and the rapidly changing landscape of genetic/genomic testing, we suggest contacting a genetics expert for advice regarding follow-up testing options.

### Technological and logistical test limitations

The current gold standard for paternity testing involves querying 15 to 20 STRs, using simple PCR technology developed in the late 1980s. Most labs prefer buccal samples, and not all labs may accept semen samples, which would be required in the case of male chimeras. STR-based testing of semen DNA can also fail due to insufficient sensitivity of the test, if the minor genome is rare (less than ~ 5%), in that tissue sample.

Interestingly, one exception is with non-invasive prenatal paternity testing, which utilizes SNP-based microarray on a sample of the mother’s blood. This test, we surmise, may have similar detection rates to ancestry testing if the chimeric parent’s minor genome is present at detectable levels in cell-free fetal DNA and *if* the lab reports second degree relationships.

Diagnosing a mother with tetragametic chimerism may be substantially more difficult, especially if the minor genome is not present in cervical cells or cells from other easily accessible tissues. Two highly publicized cases involving mothers with tetragametic chimerism, Karen Keegan and Lydia Fairchild, required additional molecular genetic testing to confirm their diagnoses and resolve issues surrounding maternity. Keegan’s condition was diagnosed by extracting DNA from a thyroid tissue sample [11], whereas Fairchild’s condition was diagnosed through DNA derived from a cervical test [12]. For females, procuring and testing gametes may involve substantially more complex processes.

SNP-based microarrays also have limitations for conclusively identifying someone as a chimera. The lower limits of detection of chimeras using this particular SNP platform are currently unknown. We believe the sensitivity of SNP-based arrays in detection of tetragametic chimerism is similar to that of somatic mosaicism, if not better. Conlin et al. were able to detect a chimeric XX/XY individual using SNP-array technology. The sample was estimated to have between 20 and 45% chimeric cells present. Conlin et al. predict SNP-based assays to be capable of detecting samples with as low as 5% chimerism [13].

Genetic testing is evolving rapidly. Costs of more robust platforms such as Next Generation Sequencing are dropping equally quickly. In the near future, single cell sequencing may become more affordable and accessible to parental DNA testing clients who require follow-up testing. For additional current information, we recommend contacting a genetic specialist with expertise in DNA relationship testing.

### DNA relationship laboratory test reporting and legal recommendations

Considering the serious implications of false negative paternity results, there is a need for a more rigorous system that anticipates chimeras and provides safeguards against false negative test reporting. Currently, the American Association of Blood Banks (AABB) Relationship Testing Program Unit does not specify recommendations about chimeras for laboratories that offer relationship DNA testing in the USA. Although the AABB does establish thresholds, cutoffs for inclusion/exclusion differ from laboratory to laboratory [1], interpretation may be subjective, human errors are possible, and genetic anomalies could impact the conclusions.

We recommend that relationship DNA testing laboratories inform clients of the residual risk of false negative results due to genetic inconsistencies such as chimerism, ideally during informed consent, but certainly on any report that excludes the tested parent. Clients should be made aware that differing results may arise from different tissue samples. Clients need to be educated about inconsistencies, preferably during the process of informed consent. This way, they can be aware of when to pursue further help in case they get an unexpected negative (exclusive) result. Relationship DNA testing labs may consider running tests that assess residual Combined Paternity Index (CPI) values and annotate reports with a pattern suggestive of avuncular relationships. This information would help identify possible chimerism in the tested father and avoid false paternity exclusions resulting from undiagnosed chimerism.

If a couple disagrees with a negative paternity result of a DNA test, they may want to consider contacting a genetic expert specializing in DNA paternity testing for additional testing options.

Legal representatives that utilize relationship DNA tests need to be made aware of limitations and the differences in DNA testing methodologies. They should also be provided with guidelines for next steps if inconsistencies are suspected. [14]

In summary, a PCR-based paternity test that yields a negative result may be insufficiently sensitive to identify the second degree relationship that can occur between a parent with tetragametic chimerism and his or her child. Follow-up testing using microarray-based relationship tests and consultation with a genetics expert could help the clinic and parents to correctly identify the relationship and preserve parental rights.

### Future research

Understanding the prevalence of tetragametic chimerism can both be a study with its own academic merit and a data that would highlight the likelihood of missed paternity. Future research into large databases of SNP-microarray data and other highly sensitive DNA testing could provide a window into the prevalence within single tissue types. Single tissue tests, however, would not be sufficient in excluding chimerism from an individual, as the minor genome may be present only in other tissues. Still, research in that direction could be a good starting point to shed more light on the prevalence of chimerism.

Parental and forensic DNA testing labs might revisit cases received that were inconclusive due to contamination. Utilizing different testing methodologies, these cases may yield chimeric individuals.

Further studies of human mosaicism are warranted. Cases involving mosaicism, most notably of sex chromosomes (XX/XY), may in fact be undiagnosed chimerism.

A relevant study of chimerism to consider would be the collection of data on the prevalence of more than one genome in semen. Such a study would shed more light on the prevalence of chimerism as it pertains to paternity.

Better understanding of the detection limits of STR, SNP-microarrays and other DNA tests utilized in diagnosing and detecting chimerism is needed.

The full psychosocial impact of a diagnosis of chimerism has on the individual and their family members could not been formally studied and is another area that could benefit from research.

## Electronic supplementary material

ESM 1 (DOCX 116 kb).
