# Supplementary material for: A case of chimerism-induced paternity confusion: what ART practitioners can do to prevent future calamity for families
Source: J Assist Reprod Genet. 2017 Oct 23;35(2):345–52. doi: 10.1007/s10815-017-1064-6 (PMC5845036; doi:10.1007/s10815-017-1064-6)
Supplement: Supplementary file 1 — (DOCX 116 kb). [file 10815_2017_1064_MOESM1_ESM.docx]

Supplemental Materials

**Table S1: The Proband’s DNA Testing Outcomes**

| Tissue type | 15 STR Testing lab | Major genome (M) or  minor genome (m) |
| --- | --- | --- |
| Blood | Paternity | No Result |
| Blood | ***Forensic*** | M |
| Semen | Paternity | No Result |
| Semen | ***Forensic*** | M, m |
| Hair | Paternity | M, m |
| Nails | Paternity | M, m |
| Skin (dark) | Paternity | M, m |
| Skin (light) | Paternity | M |
| Buccal | Paternity | M |
| Buccal | ***Forensic*** | M |

*Caption:* The proband provided additional tissue samples for confirmatory testing. The major genome is defined as the chimera’s predominant genome, while the minor genome is defined as the less predominant genome, which presumably originated from the DZ twin. STR-based DNA analyses detected only the major genome in his buccal sample, blood and light toned patch of skin. Consistent with being a chimera, his hair, nails, dark toned skin patch, and semen demonstrated the presence of an alternate (minor) allele in addition to the major allele.

**Figure S1: Proband’s Semen DNA Profile**

**
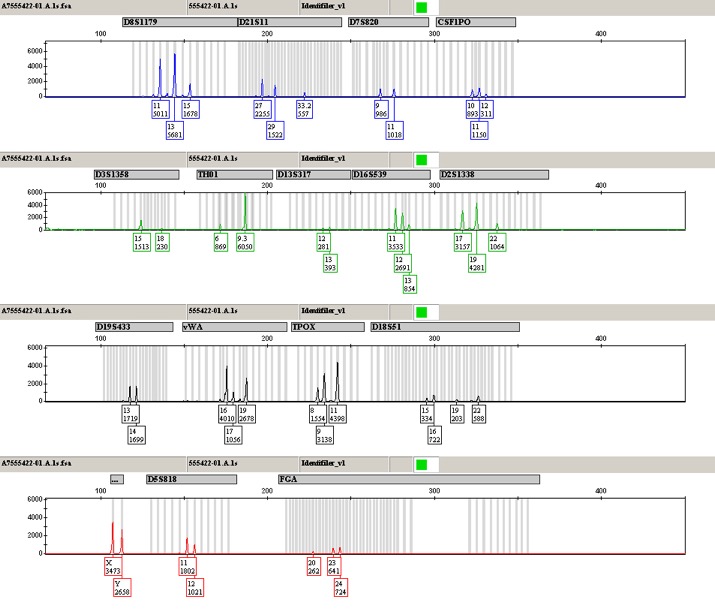
**

*Caption:* The Proband’s semen presented with 2 to 4 distinct allele sizes at each locus, indicating the presence of two sources of DNA. This sample was later confirmed to have the presence of a major and minor genome. The major genome is defined as the chimera’s predominant genome, while the minor genome is defined as the less predominant genome, which presumably originated from the DZ twin.

|  | Proband’s Mother (Buccal) | | Proband’s Father (Buccal) | | Proband (Buccal) | | Proband  (Semen) | | | |
| --- | --- | --- | --- | --- | --- | --- | --- | --- | --- | --- |
| Locus | Allele Sizes | | Allele Sizes | | Allele Sizes | | Allele Sizes | | | |
| D3S1358 | 15 | 18 | 15 | 18 | 15 |  |  | 15 | 18 |  |
| vWA | 16 | 18 | 17 | 19 | 16 | 19 | 16 | 17 | 19 |  |
| D16S539 | 11 | 12 | 11 | 13 | 11 | 12 | 11 | 12 | 13 |  |
| CSF1PO | 10 | 12 | 11 |  | 10 | 11 | 10 | 11 | 12 |  |
| TPOX | 8 | 9 | 9 | 11 | 9 | 11 | 8 | 9 | 11 |  |
| D8S1179 | 13 |  | 11 | 15 | 11 | 13 | 11 | 13 | 15 |  |
| D21S11 | 29 | 33.2 | 27 | 33.2 | 27 | 29 | 27 | 29 | 33.2 |  |
| D18S51 | 16 | 19 | 15 | 22 | 16 | 22 | 15 | 16 | 19 | 22 |
| D19S433 | 13 | 14 | 13 | 14 | 13 | 14 |  | 13 | 14 |  |
| TH01 | 6 | 9.3 | 6 | 9.3 | 9.3 |  |  | 6 | 9.3 |  |
| FGA | 20 | 23 | 24 |  | 23 | 24 | 20 | 23 | 24 |  |
| D5S818 | 11 | 12 | 11 |  | 11 | 12 |  | 11 | 12 |  |
| D13S317 | 12 | 13 | 9 | 13 | 12 | 13 |  | 12 | 13 |  |
| D7S820 | 9 | 12 | 11 |  | 9 | 11 |  | 9 | 11 |  |
| D2S1338 | 16 | 19 | 17 | 22 | 17 | 19 | 17 | 19 | 22 |  |
| Amelogenin | X |  | X | Y | X | Y |  | X | Y |  |

**Table S2: Accounting for Proband’s Alleles Utilizing His Parents**

**DDC DNA Test Report**

*Caption:* The proband’s major and minor alleles are consistent with both his mother and father. No foreign DNA is present, and we have accounted for all alleles present in his semen sample.
